# Diagnostic Pitfall in the Carotid Space: Accessory Nerve Schwannoma Simulating Cystic Metastasis—A Case Report

**DOI:** 10.3390/diagnostics16050699

**Published:** 2026-02-27

**Authors:** Roberts Tumelkans, Elza Rate, Madara Mikijanska, Can Özütemiz, Oksana Mahmajeva, Arturs Balodis

**Affiliations:** 1 Faculty of Medicine, Riga Stradins University, 16 Dzirciema Street, LV-1007 Riga, Latvia; 2 Clinic of Otolaryngology, Pauls Stradins Clinical University Hospital, 13 Pilsonu Street, LV-1002 Riga, Latvia; 3 Institute of Diagnostic Radiology, Pauls Stradins Clinical University Hospital, 13 Pilsonu Street, LV-1002 Riga, Latvia; 4 Department of Radiology, University of Minnesota, Minneapolis, MN 55455, USA; 5 Institute of Pathology, Pauls Stradins Clinical University Hospital, 13 Pilsonu Street, LV-1002 Riga, Latvia; 6 Department of Radiology, Riga Stradins University, 16 Dzirciema Street, LV-1007 Riga, Latvia

**Keywords:** schwannoma, accessory nerve, magnetic resonance imaging, carotid space

## Abstract

**Objectives**: The aim of this case report is to highlight the diagnostic challenges of carotid space masses, share clinical experience, and educate clinicians by presenting a case of a rare disease. **Introduction**: Accessory nerve schwannomas are rare, benign peripheral nerve sheath tumors. They make up only a small percentage of all cervical schwannomas. Given their rarity and varying appearance on imaging, these tumors can be difficult to accurately diagnose. Schwannomas may mimic other carotid space pathologies, such as metastatic lymphadenopathy, paragangliomas, or sympathetic chain tumors. Accurately identifying the nerve of origin before surgery is important for effective surgical planning and neurological function protection. **Case Description:** A 50-year-old woman presented with an asymptomatic left-sided neck mass. Computed tomography (CT) revealed a cystic lesion with a thick, contrast-enhancing capsule in the left carotid space, causing internal jugular vein compression and partial thrombosis. Subsequent MRI showed a 28 mm × 23 mm × 38 mm well-defined mass with characteristic schwannoma features, including T2/Short tau inversion recovery (STIR) hyperintensity, peripheral enhancement, central cystic degenerative components, and peripheral diffusion restriction with corresponding lower apparent diffusion coefficient (ADC) values. Split-fat sign and fascicular sign were also seen on the MRI. Despite these imaging findings, the radiological interpretation suggested a sympathetic chain schwannoma as the most likely diagnosis. The correct diagnosis of accessory nerve schwannoma was established intraoperatively when the mass was visualized to be attached to the accessory nerve. **Conclusions**: This case highlights that even with suggestive MRI features, the rarity of accessory nerve schwannomas can lead to misidentification of the nerve of origin. Accurate diagnosis may require intraoperative visualization, thus marking the importance of including accessory nerve involvement in the differential diagnosis of carotid space masses.

## 1. Background

Schwannomas are benign, well-circumscribed, slowly growing tumors arising from the nerve sheath, which are encountered in approximately 5% of soft tissue neoplasms [1]. Accessory nerve schwannomas are very rare. Of all schwannomas of various nerve structures located intracranially and extracranially, approximately 45% are found in the neck region, of which accessory nerve schwannoma accounts for only 4% of cases, compared to the more commonly seen vagus nerve and sympathetic chain ganglia [2]. In a study of the localization of accessory nerve schwannomas in the head and neck region, including 63 patients, extracranial localization was observed in only 14.3% of cases [3].

The clinical manifestations of accessory nerve schwannomas depend on their location. Clinical symptoms are more commonly observed in intracranial and cervical spinal canal schwannomas, whereas extracranial schwannomas are often asymptomatic and present as neck masses.

Since other pathologies may present with similar clinical manifestations, several differential diagnoses should be considered in cases of neck masses, such as malignant nerve tumors, metastatic lymphadenopathy, lymphomas, and paragangliomas [4].

Magnetic resonance imaging (MRI) is considered to be the best diagnostic method. The characteristic MRI findings of these tumors are an isointense or hypointense signal on T1, notable enhancement on contrast-enhanced T1-weighted images, and a heterogeneously hyperintense signal on T2 with hemosiderin presence in larger tumors. Several other findings, such as the target sign, split-fat sign, and fascicular sign, are described [5,6]. Precise identification of anatomical structures, such as associated nerves and adjacent blood vessels, is of utmost importance prior to surgery, thereby reducing diagnostic errors, surgical complications, and outcomes.

These tumors have characteristic histological features described as Antoni type A and type B tissue. Type A has high cellularity and a well-organized structure, elongated nuclei in palisading patterns, strong S-100 protein positivity, and Verocay bodies, whereas type B tissue has low cellularity, a less organized and degenerative structure, myxoid, and edematous stroma [3].

In cases of accessory nerve schwannomas, the treatment of choice is surgery, with the goal of achieving as complete a tumor resection as possible while preserving nerve function [7].

We present a clinical case of a rare pathology—an accessory nerve schwannoma in which the correct diagnosis was established intraoperatively, as the preoperative MRI interpretation suggested a lesion arising from the sympathetic chain.

This case demonstrates an incorrect identification of the nerve of origin. Therefore, we wish to emphasize the importance of precise anatomical topography of the nerves, their associated masses, and the blood vessels for accurate diagnosis. We also aim to draw attention to the MRI features of accessory nerve schwannoma with cystic degeneration.

## 2. Case Report

A 50-year-old woman first presented to the doctor with a complaint of a left neck mass. During examination, a painless, soft, mobile left neck mass was found next to the sternocleidomastoid muscle. No previous medical history was noted.

**Figure 1:**
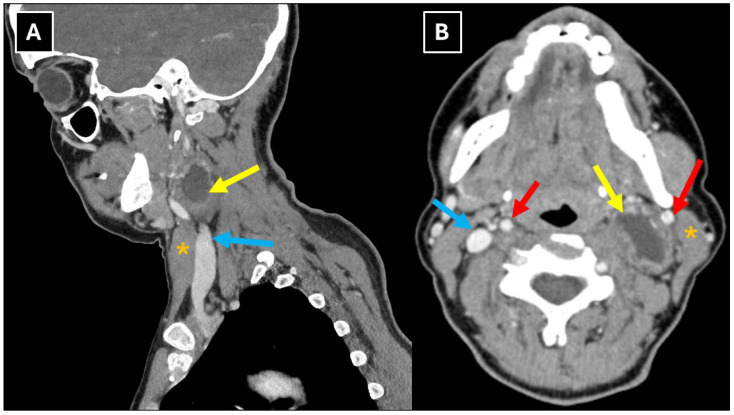
Contrast-enhanced neck CT with soft tissue window in sagittal plane (**A**) and axial plane (**B**) shows a left carotid space mass with internal cystic appearance (yellow arrows) and a thick, contrast-enhancing capsule, located medially to the sternocleidomastoid muscle at the C3-C4 level. The internal jugular vein is compressed. Red arrows—internal carotid artery, blue arrows—internal jugular vein, orange asterisks—sternocleidomastoid muscle.

Contrast-enhanced neck CT revealed a cystic mass with a thick, contrast-enhancing capsule in the left carotid space, compressing the jugular vein (Figure 1). The internal jugular vein was partially thrombosed proximally and distally to the mass and completely compressed at the level of the mass. No enlarged lymph nodes or changes in the surrounding tissues and organs were detected. The patient was referred to the otolaryngology department for further evaluation and surgical treatment.

On physical examination by ENT, a painless, soft, mobile mass was palpated under the left mandibular angle, behind the sternocleidomastoid muscle. No enlarged lymph nodes were palpated in the other neck regions. The remaining ENT system exam was otherwise unremarkable. Biochemical tests did not reveal any abnormality.

**Figure 2:**
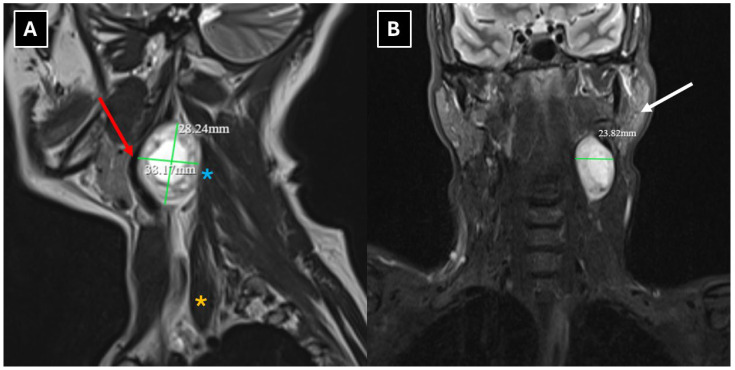
MRI scan of the neck. (**A**): Sagittal T2-weighted and (**B**): coronal STIR images show a well-defined mass located in the dorsal part of the carotid space at the C2-C4 level, with a ventral displacement of the internal and external carotid arteries. The mass measured 28 × 23 × 38 mm (AP × LL × CC). The mass demonstrates heterogeneous intermediate intense appearance, with peripheral and central areas of marked hyperintensity, suggestive of a peripheral solid component and central cystic degeneration. Red arrow—internal carotid artery; white arrows—parotid gland; orange asterisk—sternocleidomastoid muscle; blue asterisk—middle scalene muscle.

**Figure 3:**
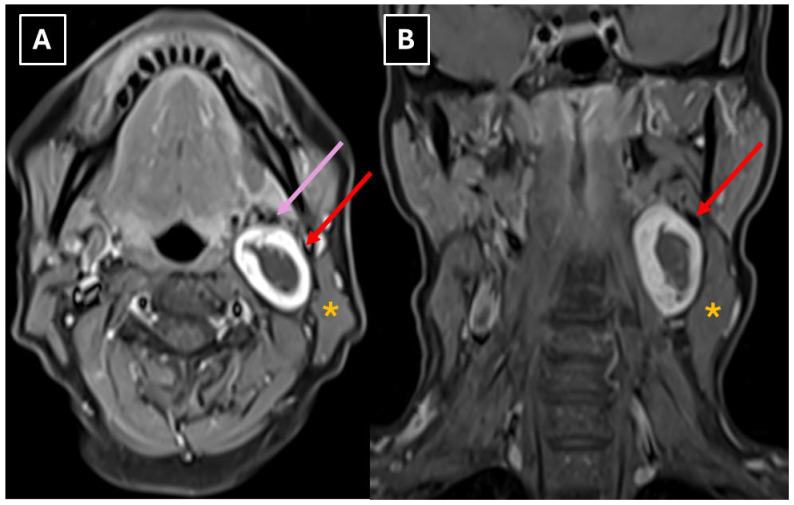
Post-contrast MRI scan of the neck. (**A**): Axial T1-weighted TSE Dixon (water-only) and (**B**) coronal T1-weighted TSE Dixon (water-only) show predominantly low-to-intermediate signal intensity with a thick, smoothly marginated peripheral rim of avid contrast enhancement and a central non-enhancing, hypovascular hypointense area—a destructive cavity, consistent with cystic degenerative components. Red arrow—internal carotid artery; pink arrow—external carotid artery; orange asterisk—sternocleidomastoid muscle.

**Figure 4:**
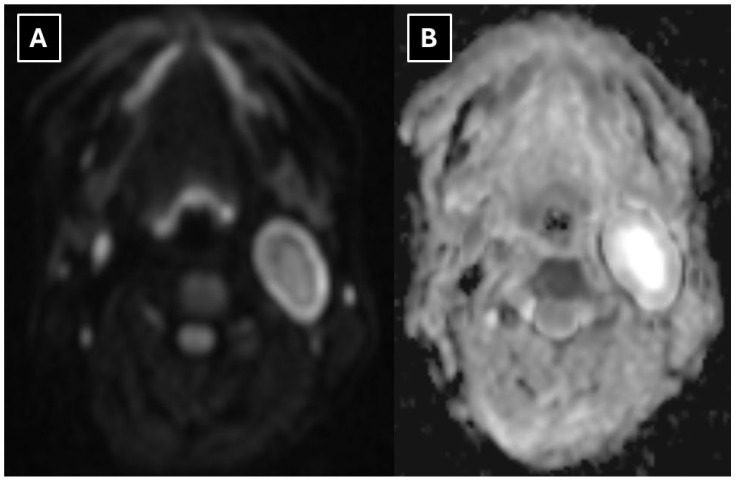
MRI scan of the neck. (**A**) Axial TRACE map of DWI b1000 and (**B**) corresponding apparent diffusion coefficient (ADC) map show mild diffusion restriction in the solid peripheral components and facilitated diffusion internally, secondary to cystic degeneration. The diffusion pattern is suggestive of a benign nerve sheath tumor.

A neck 1.5 T MRI revealed a 28 × 23 × 38 mm (AP × LL × CC) well-defined mass in the dorsal part of the left carotid space, displacing the external and internal carotid arteries ventrally (Figure 2). The mass appeared heterogeneous on the STIR sequence with a markedly high signal. It also showed intense peripheral contrast enhancement and a central cystic degenerative cavity (Figure 3). On DWI, the diffusion was facilitated internally, and the peripheral solid-enhancing component demonstrated intermediate diffusion reduction (Figure 4). On MRI, there was no evidence of aggressive extracapsular infiltration of adjacent soft tissues and blood vessels. While a few tiny lymph nodes were observed posterior to the lesion, measuring up to 5 mm in transverse diameter, these were elongated in shape and without pathological features.

The radiologist concluded that a sympathetic chain schwannoma was the most likely diagnosis, given that the lesion was localized between the external and internal carotid arteries.

Based on clinical and radiologic findings, a medical council concluded that the mass was most likely benign and decided to proceed with surgery as the primary treatment. The patient underwent surgical excision of the mass under general anesthesia. During the procedure, a smooth, cystic mass was visualized, attached to the accessory nerve. The mass was separated from the surrounding tissues and the nerve. To identify the nerve, anatomical landmarks were used based on the entry of the accessory nerve into the sternocleidomastoid muscle, 2–3 cm below the mastoid process. Its downward course along this muscle and its proximity to the internal jugular vein, positioned dorsally behind the internal carotid artery, were noted. The nerve’s exit from the posterior border of the sternocleidomastoid muscle at Erb’s point was also considered, as well as the absence of a carotid sheath, which distinguishes it from other nearby nerves.

Adjacent presumed reactive lymph nodes were also excised. The surgical specimen was sent for histological examination. The postoperative period was uneventful, and the patient had no complaints. The patient was discharged in satisfactory condition without any complications.

**Figure 5:**
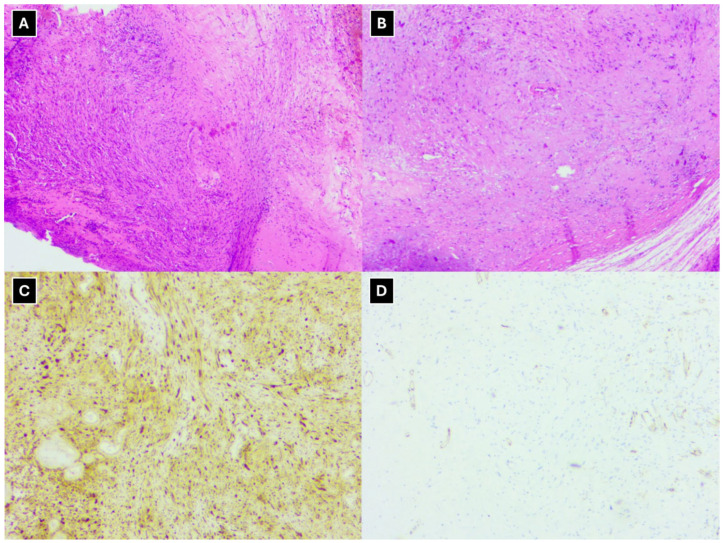
Histopathological evaluation of the schwannoma. Hematoxylin and Eosin stains in images (**A**,**B**) show a well-circumscribed, encapsulated lesion with elongated cells arranged in short bundles. Both cellular and less cellular areas of the lesion are observed. The more cellular regions display nuclear palisading, while the less cellular regions show elongated tumor cells within a myxoid stroma. The tumor cells show no signs of atypia, and no mitoses are visualized. Immunohistochemistry shows positive S-100 (**C**) and weak CD-34 positivity in a few cells (**D**).

The histopathological evaluation of the specimen showed a well-defined, encapsulated lesion composed of elongated cell proliferations in short bundles. It showed no signs of atypia or mitoses; Ki-67 was predominantly 1–2%, with focal up to 4–5% (Figure 5). Considering the morphological features and immunohistochemical results, the diagnosis was left accessory nerve schwannoma.

## 3. Discussion

Schwannomas are benign tumors arising from Schwann cells of the nerves. Schwannomas of the accessory nerve are rarely encountered, especially when they are located extracranially [8].

Similarly to this clinical case, the literature on cervical accessory nerve schwannomas mostly reports an asymptomatic course—patients notice a neck mass without other complaints [2,9,10]. In the aforementioned study, only one of nine patients with extracranial schwannoma localization reported pain in the mass region [3]. Neurological symptoms are observed mainly in cases of intracranial and cervical spinal canal localization [3].

Both the clinical presentation and the presence of a solitary node in the carotid space may pose diagnostic challenges. In clinical case reports, authors mention clarification of the diagnosis during surgery and by histological examination of the lesion [1,3,11,12]. Usually, when such a clinical picture is observed, primary hypotheses include lymphadenopathy, metastatic disease, or other more common lesions. Clinical cases have been described in the literature in which, based on objective findings (a solitary painless mass on the lateral surface of the neck) and CT results (an oval hypodense mass effect with necrosis and calcifications), the primary diagnosis was lymphadenopathy or a solitary metastasis; a significant role in verifying the correct diagnosis was played by intraoperative findings and histological examination of the material, proving accessory nerve schwannoma [10].

Although malignant peripheral nerve sheath tumors (MPNST) are rarely seen in the neck, given the observed diffusion restriction (Figure 4), these tumors should be considered in the differential diagnosis. This includes neurogenic sarcomas, malignant schwannomas, and neurofibrosarcomas [13,14]. About half of these tumors are associated with neurofibromatosis type 1 [14,15]. At disease onset, the first symptom is the appearance of a mass; however, unlike schwannomas, this is followed by rapid, aggressive tumor growth and a relatively early onset of other symptoms due to compression of surrounding tissues [14,15]. MRI features of MPNST include a significantly greater mass effect, cystic changes within the tumor, a perifocal edema-like zone, and peripheral contrast enhancement [16,17]. The use of DWI sequences, applying ADC values as a biomarker, is important in the differential diagnosis of schwannomas from malignant peripheral nerve sheath tumors [18]. The rationale for relying on these indicators is their high diagnostic accuracy in terms of both method specificity and sensitivity [19]. A criterion for malignancy in nerve tumors is diffusion restriction and a minimum ADC value of ≤1 × 10^−3^ mm^2^/s. Even greater diagnostic accuracy for MPNST is demonstrated by values with a minimum ADC of 0.89 × 10^−3^ mm^2^/s and a mean ADC of 1.15 × 10^−3^ mm^2^/s [20]. In schwannomas, high minimum ADC values > 1.1–1.2 (×10^−3^ mm^2^/s) are classically observed [21]. Therefore, a comprehensive evaluation of all MRI findings is essential for the correct identification of the pathology. In unclear cases, histopathology and immunohistochemistry play a crucial role.

In this case, the mean ADC value in the central part of the mass was 2.8 × 10^−3^ mm^2^/s, but in the peripheral part, it was 1.8–1.9 × 10^−3^ mm^2^/s. Despite the diffusion restriction seen in Figure 4, the ADC map values did not decrease significantly either centrally or peripherally, falling outside of the previously specified malignancy criteria, indicating a benign tumor.

Metastatic lesions of the carotid space also pose a diagnostic challenge, especially in cases where a primary tumor has not been diagnosed. Metastasis to cervical lymph nodes is characteristic of carcinomas of various localizations, such as breast, lung, pharynx, and thyroid [11]. In imaging, their differentiation is aided by their effect on adjacent structures and their position relative to the carotid arteries. Metastatic lymphadenopathy is variable and does not show the characteristic effect on the carotid artery seen in schwannomas [22]. Lymphoma often also presents as painless lymphadenopathy and is frequently localized in the neck region. CT examination with lymphoma-specific features (lymph node conglomerate) helps distinguish it from other lesions [11,22].

A similar appearance may be mimicked by endocrinologically inactive carotid body tumors—paragangliomas—whose only manifestation may be a mass in the neck region [8]. Unlike schwannomas, they are well vascularized, which helps to differentiate them using CT and MR methods. Paragangliomas show intense contrast enhancement on CT (hypervascularization) and a well-defined mass effect with a high T2 signal on MR images [11,22]. The topography of carotid space lesions also helps in differentiation. Paragangliomas are characterized by changes in the region of the carotid bifurcation, whereas in accessory nerve schwannomas, which run along the anterior aspect of the carotid sheath, the carotid arteries are displaced anteromedially [11,22].

In clinical practice, the most commonly observed vascular separation patterns in the carotid space help distinguish a sympathetic chain schwannoma from an accessory nerve schwannoma. The accessory nerve passes anteriorly through the carotid sheath. In cases of schwannoma arising from this nerve, the mass is located between the carotid artery and the internal jugular vein and most often separates the vessels, displacing the artery medially and the vein laterally [23]. The sympathetic chain is positioned posteromedially to the carotid artery, and in cases of schwannoma, it displaces the carotid artery and the jugular vein together anteriorly and laterally without separating them [9,23,24]. Other atypical vascular displacement patterns are encountered less frequently; however, in clinical diagnosis, the relationship of the mass to the vessels is far more important than the displacement itself [23]. We wish to emphasize the importance of this fact for accurate nerve identification because, in the presented case, insufficient attention was paid to the spatial relationship between the mass and the blood vessels.

Localized neurofibromas may also resemble schwannomas, but they are more closely associated with the affected nerve and characteristically grow more longitudinally. In imaging, their relationship with the affected nerve helps to differentiate them [11].

**Figure 6:**
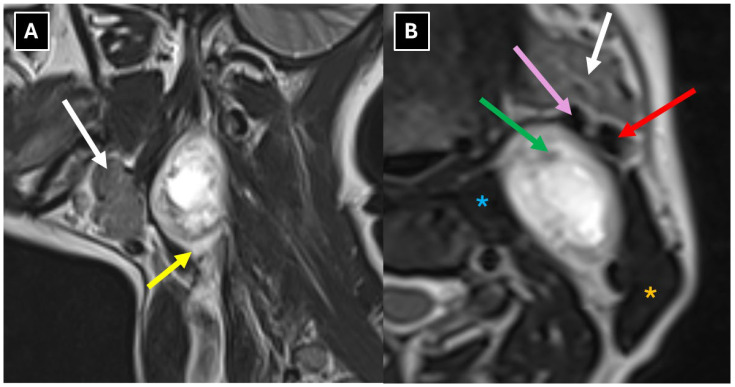
MRI scan of the neck. (**A**) Sagittal T2-weighted sequence shows a hyperintense, thin peripheral fat rim representing the “split-fat sign”. (**B**) Axial T2-weighted TSE Dixon (in) shows a round hypointense structure with a peripheral high signal—“fascicular sign”. Yellow arrow—“split-fat sign”; green arrow—“fascicular sign”; white arrows—parotid gland; red arrow—internal carotid artery; pink arrow—external carotid artery; orange asterisk—sternocleidomastoid muscle; blue asterisk—longus.

The main diagnostic methods for accessory nerve schwannomas are CT of the neck soft tissues with intravenous contrast enhancement and magnetic resonance imaging of the neck soft tissues with contrast enhancement [2,12]. In some clinical case reports, ultrasound and fine-needle aspiration (FNA) biopsy were used first in primary diagnostics, followed only later by CT or MRI [2,12]. Many authors consider the FNA procedure to be unhelpful [9]. A pathognomonic sign of schwannomas in CT is the presence of a high-density rim around a low-density lesion [3]. In MR examinations, schwannomas are characterized by low T1 signal intensity and high T2 signal intensity with pronounced contrast enhancement [11,22], though the contrast enhancement is higher in Antoni type B tissue than type A. There is a known correlation between histological and MR imaging findings, which is determined by the morphological structure of the tissue. Accessory nerve schwannoma with Antoni type A tissue is characterized by increased cellularity and nuclear palisading, which on MRI appears as hyperintensity on T1W, as well as more uniform contrast enhancement [5,25]. Antoni type B tissue is characterized by a low number of cells, with a predominance of myxomatous stroma and cystically altered areas, which in turn are characterized by heterogeneity on MRI images [5,26]. This type is characterized by T1W isointensity and hyperintensity on T2W and contrast-enhanced T1W. Consequently, a heterogeneous tumor seen on MR images histologically shows a predominance of Antoni type B tissue, and this correlation is associated with cystic degeneration of the tumor and hemosiderin deposition [5,25,26]. In this case, Antoni type A tissue primarily made up the peripheral part of the mass, which correlates with the uniformly hyperintense contrasted rim seen in Figure 3. Additionally, the increased cellularity of Antoni type A tissue presented as peripheral diffusion restriction seen in Figure 4. However, Antoni type B tissue was observed in the central destructive cystic cavity, which corresponded to the hyperintense central part of the mass seen in Figure 2 and Figure 6, as well as the hypointense zone in Figure 3.

There are other characteristic MRI features that can help identify schwannomas, such as the split-fat sign, fascicular sign, and target sign. The split-fat sign has a thin perifocal fat tissue rim in non-fat-suppressed sequences and was observed in this case (Figure 6). The fascicular sign presents with multiple small round structures with a peripheral hyperintense signal, which was also observed in this case (Figure 6). Extracranial schwannomas exhibit the target sign more frequently than intracranial schwannomas, although it was not present in this case. The target sign is characterized by central hypointensity and peripheral hyperintensity on T2-weighted images.

In this case, the patient first underwent a CT examination of the neck soft tissues with intravenous contrast administration, which revealed a well-defined cystic lesion on the left side of the neck, posterior to the sternocleidomastoid muscle, with a thick, contrast-enhancing capsule (Figure 1). The radiological conclusion was a parapharyngeal neoplasm with an effect on the carotid arteries and the jugular vein. In some cases of accessory nerve schwannoma described in the literature, CT images showed a well-defined, hypodense, non-contrast-enhancing lesion [2], but in others, an isodense lesion with hypodense areas, regions of necrosis, and calcifications [10,12]. It should be noted that in this clinical case, CT examination revealed an effect on the internal jugular vein, compressing it and resulting in partial venous thrombosis, which was clinically asymptomatic. Analysis of the available literature showed that only one case reported CT findings indicating involvement of the internal jugular vein [12].

The MRI method provides relatively accurate diagnostics, and many authors consider it superior at differentiating the nerve from which the lesion originates [10]. Both in this case and in schwannoma cases described in the literature, MRI demonstrates characteristic features such as an isointense signal on T1 (Figure 3), a hyperintense and heterogeneous signal on T2 (Figure 2 and Figure 4), intense peripheral contrast enhancement, often central hypodensity, a high signal on diffusion sequences, a high ADC, and lower values peripherally [2].

However, as demonstrated by this clinical case, the tumor type—schwannoma—was accurately determined, but the etiology was not precisely identified. Initially, it was associated with a schwannoma of the sympathetic chain rather than the accessory nerve. This can be explained by the extreme rarity of this pathology, which affects the radiologist’s ability to very precisely recognize schwannomas and the relationships between the nerve itself and the vascular structures. The literature also contains data on preoperative diagnostic difficulties in cervical schwannomas—the correct nerve pathology was identified in 83% of cases [9].

This clinical case also demonstrates histological changes consistent with schwannoma in the surgical material, with cells of a specific nature, histochemically highlighting positive S-100 protein as a specific schwannoma biomarker, which is also noted by other authors [3,9,12,27].

In cases of schwannoma, the treatment of choice is surgery. In this case, surgery was performed with a preoperative radiological diagnosis most likely indicating a sympathetic chain schwannoma; however, intraoperatively, the lesion’s association with the accessory nerve was confirmed. Successful separation of the schwannoma from the nerve and its resection were performed without causing postoperative complications—weakness of the trapezius muscle and sternocleidomastoid muscle. The literature describes cases with complications in which weakness of the corresponding muscles persisted for several months after surgery [2].

## 4. Conclusions

In cases of a lateral neck mass, radiologists should consider the possibility of a schwannoma arising from the cervical nerves, including the accessory nerve. MRI must accurately determine the location of the nerves, blood vessels, and surrounding tissue, as well as the tumor’s relationship to these structures. Evaluation of MRI signal intensity and contrast enhancement patterns plays a significant role in the diagnostic process. Accurate preoperative diagnosis allows surgeons to choose the appropriate surgical strategy, which preserves nerve function as much as possible. The most optimal treatment outcomes are ensured through strong interdisciplinary collaboration between radiologists and surgical teams.

## Data Availability

The original contributions presented in this study are included in the article. Further inquiries can be directed to the corresponding author.
